# Evaluation of retinal structural and functional changes after silicone oil removal in patients with rhegmatogenous retinal detachment: a retrospective study

**DOI:** 10.1186/s40942-023-00519-z

**Published:** 2024-01-02

**Authors:** Ran Dou, Rui Li, Rui-chan Li, Yan-ru Yu, Jin-xiu Zhou, Rui-mei Li, Xia-ping Wang, Dong-chang Zhang, Jian Jiang, Song Chen

**Affiliations:** 1 Department of Ophthalmology, Qilu Hospital (Qingdao), Cheeloo College of Medicine, Shandong University, 758 Hefei Road, Qingdao, 266035 China; 2 Shanxi Aier Eye Hospital, Taiyuan, 030000 Shanxi Province China; 3 Clinical College of Ophthalmology, Tianjin Eye Hospital, Tianjin Medical University, No.4 Gansu Road, Heping District, Tianjin, 300020 China

**Keywords:** Microcirculation, Microperimetry, Optical coherence tomography angiography, Rhegmatogenous retinal detachment, Silicone oil

## Abstract

**Background:**

To evaluate retinal structural and functional changes after silicone oil (SO) removal in eyes with macula-off rhegmatogenous retinal detachment (RRD).

**Methods:**

Best-corrected visual acuity (BCVA) testing, microperimetry, and optical coherence tomography angiography were performed in 48 eyes with macula-off RRD before and 3 months after SO removal. The values of healthy contralateral eyes were used as control data. Correlations between retinal vessel density (VD), retinal nerve fiber layer thickness (RNFLT), the interval between retinal detachment and surgery, the duration of SO tamponade, the follow-up time after SO removal, and visual function were analyzed.

**Results:**

Significant increases in 2˚ fixation rate (FR), 4˚ FR, 2˚ mean retinal sensitivity (MRS), 6˚ MRS, parafoveal superficial capillary plexus VD and RNFLT were observed after SO removal (all *P* < 0.05). The increase of 2˚ MRS and 6˚ MRS were correlated with the duration of SO tamponade and the follow-up time after SO removal respectively (all *P* < 0.05). The last 2˚ MRS and 6˚ MRS were correlated with the duration of SO tamponade, the interval between retinal detachment and surgery, and the follow-up time after SO removal (all *P* < 0.01). The last FR in RRD eyes was close to that of contralateral eyes (*P* > 0.05).

**Conclusion:**

Retinal structure and function improved to different degrees after SO removal. Fixation stability and retinal sensitivity increased more than BCVA postoperatively. Retinal sensitivity, which was affected by the interval between retinal detachment and surgery and the duration of SO tamponade, gradually recovered after SO removal.

## Background

Silicone oil (SO) -related vision loss (SORVL) is a side effect of SO which is frequently utilized in rhegmatogenous retinal detachment (RRD). Best corrected visual acuity (BCVA) is not the only assessment used to evaluate postoperative visual function. Unlike the BCVA test, which depends on the ability to distinguish spatial patterns, retinal sensitivity tested using microperimetry is based on the ability to discriminate low-contrast signals [1]. Few studies have investigated the effect of SO tamponade on retinal sensitivity. Previous studies have shown that retinal sensitivity decreased after SO tamponade and increased after SO removal in RRD eyes [2, 3]. However, the underlying mechanisms remain unclear. Our previous studies showed that SO tamponade could result in a decrease in macular and peripapillary capillary vessel density (VD) and retinal nerve fiber layer thickness (RNFLT) [4, 5]. Whether the effect of SO on retinal sensitivity is correlated with VD and RNFLT remains unclear.

This study aimed to evaluate retinal structural and functional changes in RRD eyes before and 3 months after SO removal using microperimetry and optical coherence tomography angiography (OCTA), to analyze related correlation.

## Methods

This retrospective study was performed in accordance with the tenets of the Declaration of Helsinki and was approved by the ethics committee. Unilateral RRD eyes successfully repaired using pars plana vitrectomy (PPV) with SO tamponade were followed-up for more than 3 months after simple SO removal surgery from January 2020 to December 2021. All participants underwent examinations before and 1 week, 2 weeks, 1 month, 3 months, and 6 months after SO removal, including BCVA, intraocular pressure (IOP), slit-lamp biomicroscopy examination, fundus examination. OCTA and microperimetry were performed once more than 3 months after SO removal.

The exclusion criteria were as follows: (1) age < 18 years; (2) prior ocular surgery; (3) additional ocular diseases (e.g., glaucoma, uveitis, retinal vascular disease, optic disc abnormalities, or macular diseases in either eye); (4) postoperative complications (e.g., endophthalmitis, vitreous hemorrhage, or retinal redetachment); (5) axial length ≥ 26.5 mm, or an axial length difference of > 0.3 mm between both eyes; (6) any medical condition that could affect the hemodynamics of the eye (e.g., hypertension, diabetes, or Alzheimer’s disease); and (7) refractive medium opacity.

Simple SO (Oxane 5700, Bausch & Lomb, Rochester, NY, USA) removal surgery was performed by the same surgeon using the Alcon Constellation system (Alcon, Fort Worth, TX, USA). Internal limiting membrane (ILM) peeling, scleral buckling, and perfluorocarbon use were avoided in all patients during both surgeries. Fluid-air exchange was applied to reduce the number of emulsified SO droplets. The scleral incisions were sutured using 8–0 absorbable sutures. Phacoemulsification and implantation of foldable intraocular lenses were performed (when required) prior to PPV or SO removal during both surgeries. Approximately 4-mm was removed from the central part of the posterior capsule to prevent opacification.

**Fig. 1:**
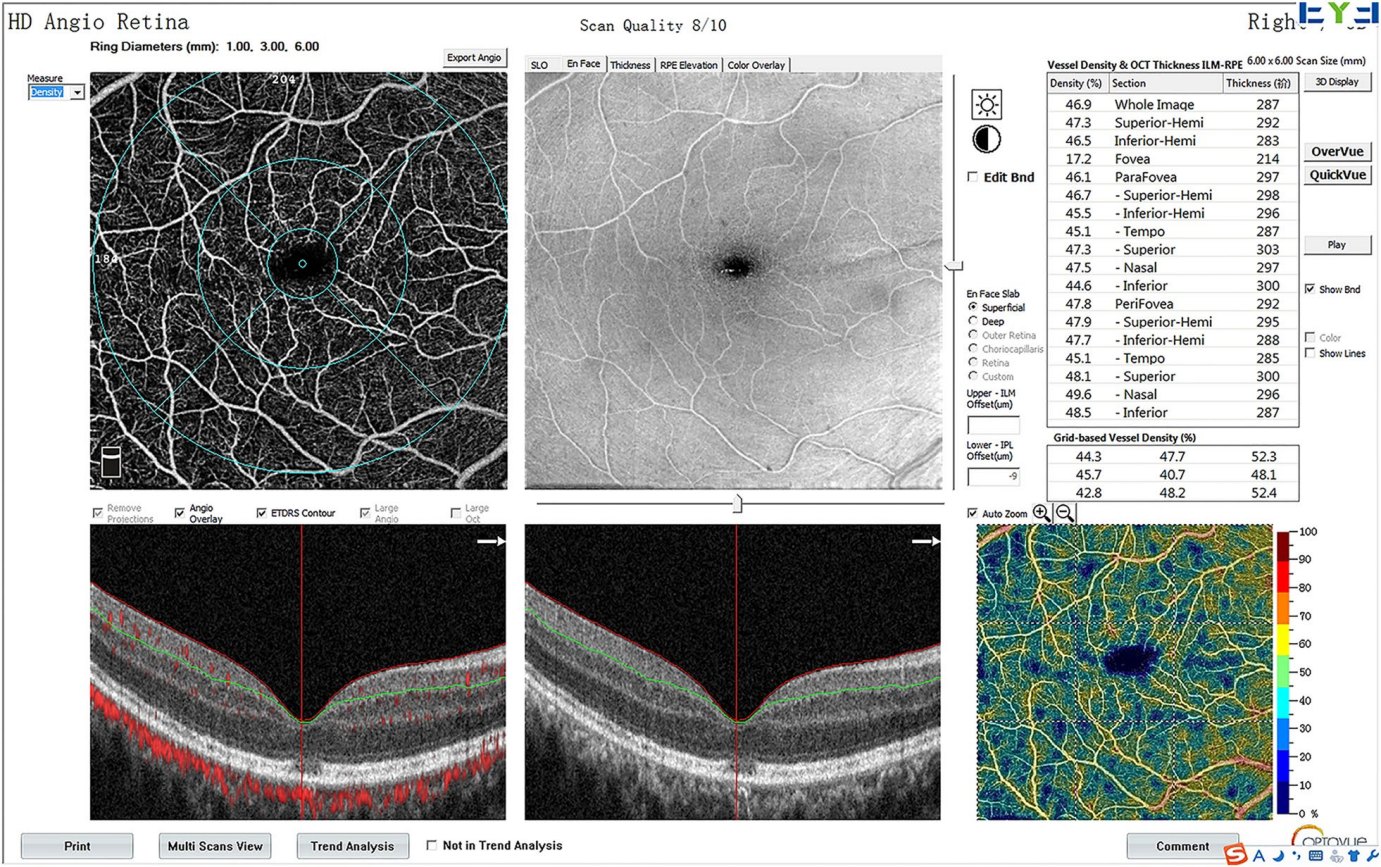
Example of OCTA image automatically generated showing superficial enface scan, B-scan, and values of vessel density and thickness

OCTA was performed to evaluate the parafoveal VD using the RTVue XR Avanti AngioVue system (Optovue Inc, Fremont, CA, USA) with AngioVue OCTA software (version 2018.1.0.43). Relative data were obtained using a split-spectrum amplitude-decorrelation angiography algorithm. A three-dimensional projection artifact removal technique was applied to improve the accuracy of the data. The definitions of the parafoveal region, superficial capillary plexus (SCP) VD, and deep capillary plexus (DCP) VD were consistent with those in the literature [4]. The parafoveal SCP VD, DCP VD, RNFLT, foveal macular thickness (FMT), and foveal avascular zone (FAZ) were automatically generated (Fig. 1).

**Fig. 2:**
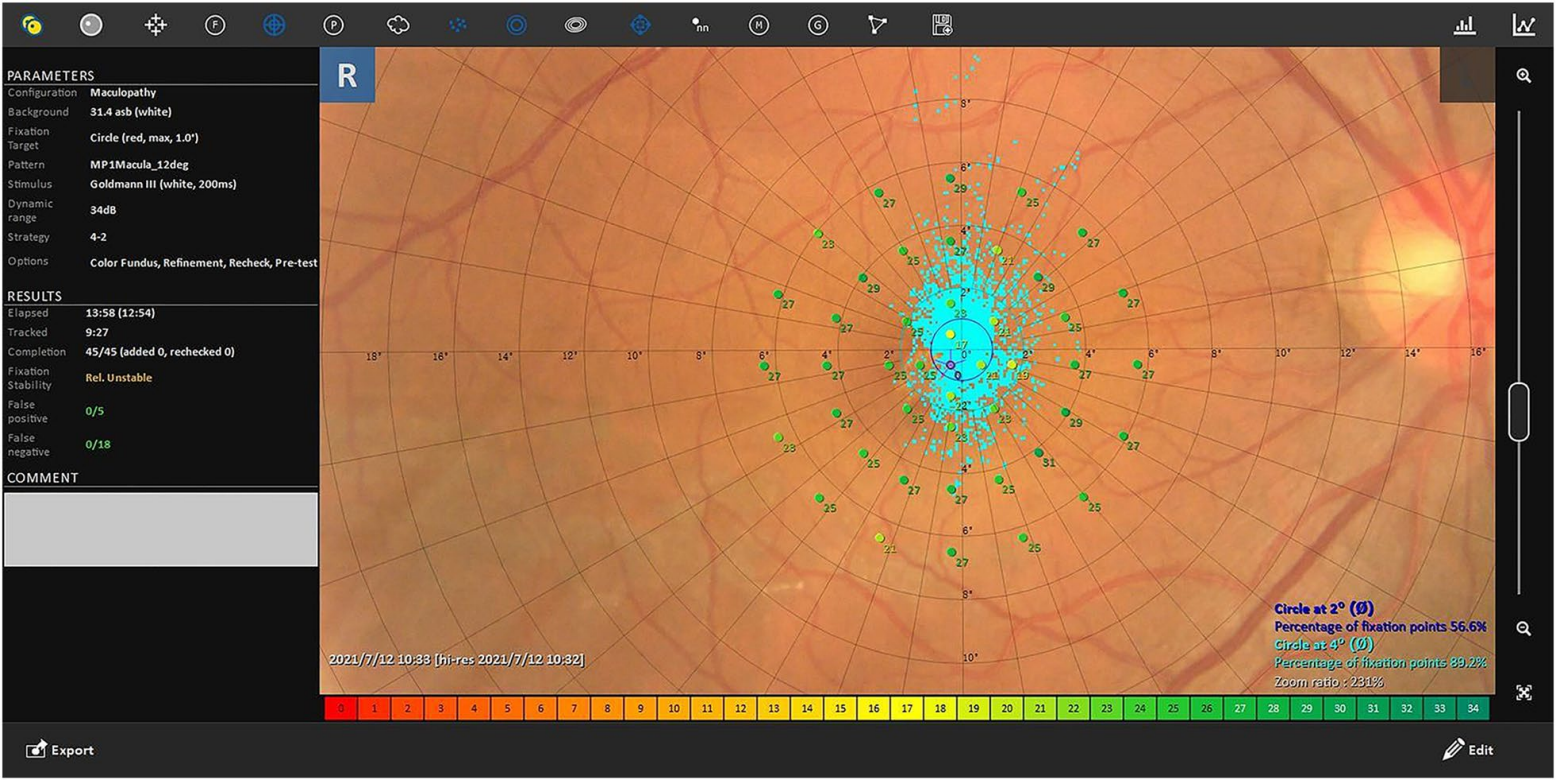
Example of microperimetry assessment image automatically generated showing retinal sensitivity of each point, 2˚ fixation rate and 4˚ fixation rate

Microperimetry assessment was performed to evaluate the fixation rate (FR) and mean retinal sensitivity (MRS) using microperimeter-3 (MP-3) (Nidek, Aichi, Japan) in a dimly lit room. Two experienced operators performed the examination. All patients had a pupil size > 4 mm in diameter. The MP-3 measurement was carried out using a 4–2 staircase strategy with a Goldmann III stimulus size. The 45 test points were measured accordingly. The maximum luminance of MP-3 was 10,000 asb and the stimulus dynamic ranged from 0 to 34 dB. The fixation target was a 1˚-diameter red circle, and the background luminance was set at 31.4 asb. Only reliable visual functions, defined as those with < 15% false positives and false negatives, were used in the analyses. Using the obtained retinal sensitivities, 2˚ MRS, 6˚ MRS, superior 6˚ MRS, and inferior 6˚ MRS were calculated. 2˚ FR and 4˚ FR were automatically generated (Fig. 2). The same variables in the healthy contralateral eyes were used as control data.

### Statistical analysis

All results were analyzed using SPSS 20.0 for Windows (SPSS, Chicago, IL, USA) software. BCVA was converted to the logarithm of the minimum angle of resolution units. Differences between pre- and post-operative values and between the RRD and healthy contralateral eyes were compared using a Wilcoxon signed- rank test. Correlations were evaluated using a Spearman test. Statistical significance was set at *P* < 0.05.

## Results

Table 1 shows the baseline demographics and clinical characteristics of the 48 study patients. Phacoemulsification and foldable IOL implantation were performed in 13 eyes during PPV with SO tamponade surgery and 10 eyes during SO removal surgery. The duration of all SO removal surgeries was less than 60 min. A transient increase or decrease in IOP occurred within 1 month after SO removal surgery, after which IOP returned to normal. The increase in IOP was controlled with topical antiglaucoma medication.

**Table 1:** Demographics and baseline characteristics of the included patients (n = 48)

Variable	Value (with range)
Mean age (years)	51.9 ± 13.4 (22–75)
Sex (male/female)	22/26
RRD eye (right/left)	29/19
The interval between retinal detachment and surgery (days**)**	17.4 ± 15.2 (3–75)
Mean duration of SO tamponade (months)	4.4 ± 1.2 (3–7)
Mean follow-up period (months)	4.2 ± 1.1 (3–6)

*RRD* rhegmatogenous retinal detachment, *SO* silicone oil

2˚MRS, 6˚ MRS, 2˚ FR, 4˚ FR, parafoveal SCP VD, and RNFLT increased in RRD eyes after SO removal (all *P* < 0.05). There was no significant difference in BCVA, parafoveal DCP VD, FMT, and FAZ in RRD eyes between before and after SO removal (all *P* > 0.05) (Table 2). BCVA, 2˚ MRS, 6˚ MRS, FMT, parafoveal SCP VD, and RNFLT remained lower than those in contralateral eyes after SO removal (all *P* < 0.05).

**Table 2:** Provides the preoperative and postoperative values of RRD eyes

Variable	Pre-operation	Post-operation	contralateral eye
BCVA (log)	0.67 ± 0.48	0.56 ± 0.50	0.00
2°FR (%)	72.23 ± 20.90	79.25 ± 19.06	83.63 ± 11.15
4°FR (%)	91.65 ± 11.45	94.65 ± 9.01	96.98 ± 3.02
2°MRS (dB)	19.85 ± 5.41	23.27 ± 3.64	26.33 ± 3.28
6°MRS (dB)	22.23 ± 4.27	24.75 ± 3.40	27.13 ± 2.62
Parafoveal SCP VD (%)	42.65 ± 5.46	44.96 ± 5.66	48.98 ± 5.46
Parafoveal DCP VD (%)	48.79 ± 5.02	49.83 ± 4.55	51.50 ± 5.62
Parafoveal RNFLT (µm)	299.42 ± 34.19	309.65 ± 24.27	333.65 ± 48.45
FMT (µm)	248.62 ± 59.54	244.85 ± 28.10	266.91 ± 55.94
FAZ (mm^2^)	0.32 ± 0.11	0.29 ± 0.09	0.29 ± 0.10

*BCVA* best-corrected visual acuity, *FR* fixation rate, *MRS* mean retinal sensitivity, *SCP* superficial capillary plexus, *VD* vessel density, *DCP* deep capillary plexus, *RNFLT* retinal nerve fiber layer thickness, *FMT* foveal macular thickness, *FAZ* foveal avascular zone

2˚ MRS and 6˚ MRS were correlated with the duration of SO tamponade and the interval between retinal detachment and surgery before SO removal (Table 3). 2˚MRS and 6˚MRS were correlated with the duration of SO tamponade, the interval between retinal detachment and surgery and the follow-up time after SO removal (Table 4). The increase in 6˚ MRS was correlated with that in 2˚ FR, 4˚ FR, and 2˚ MRS. The increase in 2˚ MRS was correlated with the duration of SO tamponade. The increase in 6˚ MRS was correlated with the follow-up time after SO removal (Table 5).

**Table 3:** Correlation analysis between values before silicone oil removal (Spearman method)

	Duration	Follow-up time	Detachment time	BCVA (logMAR)	2°FR	4°FR	2°MRS	6°MRS	SCPVD	DCPVD	RNFLT	FMT	FAZ
Duration	1.000												
Follow-up time	−0.330^*^	1.000											
Detachment time	0.660^**^	−0.520^**^	1.000										
BCVA	0.320*	−0.183	0.210	1.000									
2°FR	−0.070	−0.089	0.041	−0.093	1.000								
4°FR	−0.033	−0.062	0.051	−0.162	0.950^**^	1.000							
2°MRS	−0.690^**^	0.290^*^	−0.447^**^	−0.448^**^	0.092	0.058	1.000						
6°MRS	−0.764^**^	0.281	−0.517^**^	−0.436^**^	0.119	0.113	0.884^**^	1.000					
SCPVD	−0.242	0.033	−0.120	−0.083	−0.041	−0.014	0.014	0.166	1.000				
DCPVD	−0.261	−0.024	−0.162	−0.320^*^	−0.022	−0.066	0.271	0.304^*^	0.217	1.000			
RNFLT	−0.301^*^	−0.002	−0.235	−0.252	0.085	−0.004	0.318^*^	0.284	−0.017	0.234	1.000		
FMT	−0.182	−0.098	−0.202	−0.109	0.035	−0.057	0.154	0.165	−0.156	0.077	0.794^**^	1.000	
FAZ	0.078	0.203	0.138	−0.041	0.234	0.239	0.020	−0.055	0.271	0.039	−0.095	−0.362^*^	1.000

The “Duration” represents “Duration of SO tamponade”; “Follow-up time” represents the follow-up time after silicone oil removal surgery; "Detachment time" represents the interval between retinal detachment and surgery; SCPVD, DCPVD, and RNFLT are all values of the parafoveal area

*SO* silicone oil, *BCVA* best corrected visual acuity, *FR* fixation rate, *MRS* mean retinal sensitivity, *SCP* superficial capillary plexus, *DCP* deep capillary plexus, *VD* vascular density, *RNFLT* retinal nerve fiber layer thickness, *FMT* foveal macular thickness, *FAZ* foveal avascular zone

where * represents *P* < 0.05; * * represents *P* < 0.01

**Table 4:** Correlation analysis between values after silicone oil removal (Spearman method)

	Duration	Follow-up time	Detachment time	BCVA	2°FR	4°FR	2°MRS	6°MRS	SCPVD	DCPVD	RNFLT	FMT	FAZ
Duration	1.000												
Follow-up time	−0.330^*^	1.000											
Detachment time	0.660^**^	−0.520**	1.000										
BCVA	0.224	−0.024	0.095	1.000									
2°FR	−0.255	−0.003	−0.042	−0.091	1.000								
4°FR	−0.255	−0.051	−0.102	−0.038	0.913**	1.000							
2MRS	−0.480^**^	0.494^**^	−0.832**	−0.104	−0.055	−0.025	1.000						
6MRS	−0.601^**^	0.642^**^	−0.939**	−0.131	−0.031	0.024	0.904**	1.000					
SCPVD	−0.034	−0.287^*^	−0.027	−0.108	−0.030	0.118	0.027	−0.012	1.000				
DCPVD	−0.190	−0.108	−0.131	−0.310^*^	−0.122	−0.118	0.034	0.081	0.297*	1.000			
RNFLT	−0.296^*^	−0.091	−0.268	−0.108	−0.167	−0.072	0.265	0.264	0.289*	0.343*	1.000		
FMT	−0.187	−0.166	−0.155	0.082	−0.088	−0.154	0.094	0.064	−0.204	0.084	0.547**	1.000	
FAZ	−0.080	0.134	0.016	0.017	0.070	0.118	0.063	0.089	0.071	−0.180	−0.189	−0.548**	1.000

The “Duration” represents “Duration of SO tamponade”; “Follow-up time” represents the follow-up time after silicone oil removal surgery; "Detachment time" represents the interval between retinal detachment and surgery; SCPVD, DCPVD, and RNFLT are all values of the parafoveal area

*BCVA* best corrected visual acuity, *FR* fixation rate, *MRS* mean retinal sensitivity, *SCP* superficial capillary plexus, *DCP* deep capillary plexus, *VD* vascular density, *RNFLT* retinal nerve fiber layer thickness, *FMT* foveal macular thickness, *FAZ* foveal avascular zone

where * represents *P* < 0.05; * * represents *P* < 0.01

**Table 5:** Correlation analysis between the increase of values after silicone oil removal (Spearman method)

	Duration	Follow-up time	Detachment time	BCVA	2°FR	4°FR	2°MRS all	6°MRS all	6°MRS S	6°MRS I	SCPV D all	SCPV D S	SCPV D I	DCPV D all	DCPV D S	DCPVD I	RNFLT all	RNFLT S	RNFLT I	FMT	FAZ
Duration	1.000																				
Follow-up time	−0.330^*^	1.000																			
Detachment time	0.660**	−0.520**	1.000																		
BCVA	0.038	0.170	0.048	1.000																	
2°FR	−0.111	0.031	−0.045	−0.205	1.000																
4°FR	−0.168	0.064	−0.118	−0.149	0.904**	1.000															
2°MRS all	0.302*	0.158	−0.141	−0.212	0.246	0.241	1.000														
6°MRS all	0.205	0.344*	−0.234	−0.037	0.344*	0.342*	0.898**	1.000													
6°MRS S	0.252	0.284	−0.140	0.085	0.284	0.283	0.727**	0.836**	1.000												
6°MRS I	0.137	0.313*	−0.248	−0.148	0.189	0.160	0.728**	0.815**	0.477**	1.000											
SCPVD all	0.124	−0.232	0.046	0.237	0.236	0.194	0.077	0.058	0.013	−0.038	1.000										
SCPVD S	0.131	−0.317*	0.112	0.237	0.249	0.206	0.009	−0.027	−0.021	−0.143	0.949**	1.000									
SCPVD I	0.131	−0.162	0.032	0.227	0.125	0.104	0.105	0.095	0.010	0.058	0.930**	0.786**	1.000								
DCPVD all	0.047	−0.028	−0.031	0.237	−0.162	−0.188	0.128	0.054	0.077	0.057	0.315*	0.292*	0.303*	1.000							
DCPVD S	0.115	−0.080	−0.004	0.227	−0.143	−0.184	0.136	0.046	0.125	0.027	0.289*	0.314*	0.223	0.912**	1.000						
DCPVD I	−0.008	0.065	−0.019	0.227	−0.183	−0.171	0.107	0.050	−0.014	0.074	0.234	0.146	0.298*	0.869**	0.646**	1.000					
RNFLT all	0.128	−0.163	0.074	−0.195	−0.045	−0.032	0.079	0.094	0.035	0.143	−0.198	−0.219	−0.181	−0.174	−0.071	−0.180	1.000				
RNFLT S	0.162	−0.161	0.103	−0.169	−0.059	−0.058	0.054	0.084	0.080	0.093	−0.196	−0.223	−0.169	−0.196	−0.105	−0.202	0.965**	1.000			
RNFLT I	0.059	−0.084	−0.003	−0.174	−0.045	−0.010	0.127	0.142	−0.003	0.214	−0.155	−0.186	−0.141	−0.158	−0.071	−0.123	0.949**	0.846**	1.000		
FMT	0.011	0.040	0.016	−0.195	−0.054	−0.036	0.086	0.144	0.165	0.129	−0.444**	−0.411**	−0.444**	−0.257	−0.187	−0.266	0.631^**^	0.630^**^	0.552^**^	1.000	
FAZ	−0.075	−0.126	−0.062	0.037	−0.074	0.010	0.058	−0.062	−0.019	−0.033	−0.066	0.001	−0.133	0.004	0.043	−0.057	−0.021	0.006	−0.073	−0.040	1.000

The “Duration” represents “Duration of SO tamponade”; “Follow-up time” represents the follow-up time after silicone oil removal surgery; "Detachment time" represents the interval between retinal detachment and surgery; SCPVD, DCPVD, and RNFLT are all values of the parafoveal area

*BCVA* best corrected visual acuity, *FR* fixation rate, *MRS* mean retinal sensitivity, *SCP* superficial capillary plexus, *DCP* deep capillary plexus, *VD* vascular density, *RNFLT* retinal nerve fiber layer thickness, *S* superior, *I* inferior, *FMT* foveal macular thickness, *FAZ* foveal avascular zone

where * represents *P* < 0.05; * * represents *P* < 0.01

## Discussion

Microperimetry, which has been applied in many ophthalmic disorders [6–10], can provide MRS and FR measurements for retinal function assessment. Fixation stability, which is usually a key evaluation indicator for macular hole [11], has rarely been used to evaluate visual function after RRD repair. In contrast to BCVA and MRS, there was no significant difference in 2˚ FR and 4˚ FR 3 months after SO removal between RRD and healthy contralateral eyes. Rossetti et al. [12] reported that fixation stability in macula-off RRD eyes repaired with scleral buckling recovered well after long-term follow-up. Borowicz et al. [13] evaluated retinal function in eyes with macula-on and macula-off RRD using microperimetry and found that fixation stability recovered relatively well postoperatively. Therefore, we cautiously consider that fixation stability is less affected than BCVA and MRS after experiencing macular detachment and SO tamponade.

Noda et al. [14] reported that post-operative MRS was significantly higher than pre-operative MRS in macula-off RRD eyes, and that post-operative MRS was lower in patients with pre-operative macular detachment. Borowicz et al. [13] reported a significant increase in MRS over time in eyes with macula-on and macula-off RRD after PPV and gas tamponade, and lower MRS in macula-off RRD eyes than in macula-on RRD eyes after surgery. In this study, 2˚ MRS and 6˚ MRS remained lower in RRD eyes than in healthy eyes more than 3 months after SO removal. Therefore, we suspect that detached macula may result in damage to retinal sensitivity, which is too severe to ultimately recover from.

Studies concerning the side-effects of SO have been widely reported, among which SORVL has been shown to be independent of the surgeon and the surgical procedure [2, 15, 16]. However, few studies have investigated the side-effects on retinal sensitivity and fixation stability. Several efforts were made to limit surgical factors in this study. First, the intraoperative IOP was controlled at 25 mmHg using the Alcon Constellation system. Second, simple SO removal surgery was performed in all the eyes with RRD. Complicated surgical procedures such as ILM peeling [17], stripping of the proliferative membrane, and photocoagulation were not included in this study. Third, phacoemulsification was performed prior to PPV or SO removal during both surgeries. Fourth, main outcome measures were acquired at least 3 months after SO removal. This period was sufficient for acute surgical inflammation and IOP fluctuations to subside. Through excluding the influence of surgical factors on retinal sensitivity and microcirculation, we aimed to isolate and evaluate the parameter changes due only to the SO.

In this study, we found that the degree of increase in BCVA, MRS, and FR were inconsistent after SO removal. SORVL alone cannot explain the damage to visual function caused by SO. In contrast to BCVA, statistical significance was observed in the changes in FR and MRS between before and 3 months after SO removal. Therefore, we suspect that the side effects of SO on FR and MRS are greater than those on BCVA, or that SORVL is more difficult to recover.

In addition, we found that MRS was affected by the duration of SO tamponade and the interval between retinal detachment and surgery in the state of SO tamponade and 3 months after SO removal, and recovered over time after SO removal. To date, the mechanisms underlying the side-effects of SO on retinal function remain unclear. Previous studies have found that retinal function in macula-off RRD eyes was lower than that in both contralateral eyes and eyes with macula-on RRD [18, 19]. Eshita et al. [20] performed scanning laser Doppler flowmetry to measure macular blood flow in 28 patients with macula-on RRD and found that the mean blood flow ratio in the RRD eye was lower than that in the contralateral eye, both pre- and post-operatively. Therefore, retinal ischemia at the detached areas in eyes with macula-on RRD was considered to reduce macular blood flow, which affects retinal function [18, 19].

According to the standard [10], one degree in microperimetry is equal to the length of a 250 µm fundus. Correlations between 6˚ MRS, superior 6˚ MRS, inferior 6˚ MRS, and the corresponding parafoveal SCP VD, DCP VD, and RNFLT were tested before and after SO removal, but no correlations were observed 3 months after SO removal, which indicated that retinal microcirculation was not the only factor affecting retinal sensitivity. Another study showed that outer retinal layer damage in macula-off RRD was another factor affecting visual function [21]. Similarly, no correlations were found between their increase after SO removal, which indicated that retinal microcirculation was not the only factor in the side-effects of SO on retinal sensitivity. A previous study reported that the effect of SO on fixation stability and retinal sensitivity might be related to xanthophyll pigment accumulation in the macula [22]. The interaction between lipophilic SO and pigment may also result in foveal damage, which explain the effects on fixation stability and retinal sensitivity.

Our study had some limitations. First, the retrospective single-center study design might have caused a selection bias. Second, the sample size was small and the follow-up periods were short. Third, many patients with long-term SO tamponade were excluded from this study because of poor image quality. Fourth, the effect of phacoemulsification and IOL implantation in the 10 eyes which underwent the procedure during SO removal could not be excluded. Finally, the relevant measurements of the RRD eyes before the first vitrectomy and the early period after SO tamponade were not included in the study.

## Conclusion

Retinal structure and function improved to different degrees after SO removal, and fixation stability and retinal sensitivity increased more than BCVA. Retinal sensitivity, which was affected by the interval between retinal detachment and surgery and the duration of SO tamponade, gradually recovered after SO removal.

## Data Availability

The datasets generated during and/or analysed during the current study are available from the corresponding author on reasonable request.

## Abbreviations

SO: Silicone oil
RRD: Rhegmatogenous retinal detachment
BCVA: Best-corrected visual acuity
VD: Vessel density
RNFLT: Retinal nerve fiber layer thickness
FR: Fixation rate
MRS: Mean retinal sensitivity
SORVL: Silicone oil related vision loss
OCTA: Optical coherence tomography angiography
PPV: Pars plana vitrectomy
IOP: Intraocular pressure
ILM: Internal limiting membrane
SCP: Superficial capillary plexus
DCP: Deep capillary plexus
FMT: Foveal macular thickness
FAZ: Foveal avascular zone
MP-3: Microperimeter-3
